# Physical activity in cancer care: barriers and interventions

**DOI:** 10.11604/pamj.2022.43.131.31743

**Published:** 2022-11-09

**Authors:** Youness Azemmour, Saber Boutayeb, Hassan Beddaa, Hassan Errihani

**Affiliations:** 1 Clinical Research Biostatistics and Epidemiology Laboratory, Faculty of Medicine and Pharmacy, Mohammed V University, Rabat, Morocco,; 2 Translational Oncology Research Team, National Institute of Oncology, Ibn Sina University Hospital Center, Mohammed V University, Rabat, Morocco

**Keywords:** Cancer, cancer care, healthcare professionals, physical activity

## Abstract

Cancer is a real public health problem in the world. The patients' functional cancer impact on patients needs a global care approach. Physical activity is recognized as supportive care because it benefits patients throughout the cancer care pathway. Indeed, it seems safe with scientifically proven benefits on physical functions and patients' quality of life. Promoting physical activity, asks for an improvement in the healthcare professional's knowledge and establishing rehabilitation programs through physical activity. A physical exercise program requires an assessment to adapt the program according to the patient's capacities and reactions. Physical activity interventions remain accessible to existing healthcare systems and are less costly than pharmaceutical interventions.

## Commentary

### Context

Cancer is considered a public health problem and a real burden, both individually and collectively. In 2018, estimates revealed that 18.1 million people were diagnosed with cancer, and 9.6 million died from it, making cancer the second leading cause of death worldwide [1]. The steady increase in the prevalence of cancer and the promising advances in oncological therapy have improved life expectancy. Still, the functional impact of the disease on cancer survivors remains significant. Between 1981 and 2010, the decline in the cancer mortality rate has contributed, on average, to 20% of gains in life expectancy, in populations with a high human development index [2]. However, an annual increase in both the incidence rate and cancer-related mortality was observed in the majority of African countries between 2007 and 2017 [3].

Despite the progress in oncology in saving lives, the biomedical approach alone remains insufficient to respond to the burden related to the disability situation and functional cancer impact on the cancer patient. Worldwide between 1990 and 2017, the burden of cancer was estimated at 233.5 million disability-adjusted years, of which 97% were years of life lost, and 3% were years lived with disability [3].

Otherwise, physical activity in cancer care has caused much ink to flow in the scientific literature in recent years. Some mechanisms of physical activity action on carcinogenesis have been identified, such as changes in endogenous sex, metabolic hormone levels, and growth factors [4]. Additionally, there is strong scientific evidence on the benefits and safety of physical activity in cancer care [4,5]. This evidence highlights that physical activity is increasingly becoming confirmed supportive care. However, adherence to recommended physical activity guidelines in cancer patients appears to be inadequate; on the contrary, there has been a significant regression in patients' physical activity levels shortly after diagnosis [6]. As a result, patients miss out on the physical activity benefits during and after cancer.

### Benefits and safety

Physical activity has several benefits for the patient with cancer in the all-cancer care pathway. The literature which has demonstrated the physical activity of patients with cancer is so rich that it cannot be summed up in some pages. In general, there is strong scientific evidence to support physical activity's benefits on physical functions and quality of life for the patient in all cancer care pathways. The safety of physical activity has also been supported by evidence [4].

However, some cancer side effects are wrongly considered as a barrier to physical activity promotion in cancer care. A literature review on the link between physical activity and health outcomes of cancer survivors showed that in addition to reduced risk of cancer recurrence and mortality related to cancer, physical activity appears to reduce side effects associated with cancer treatment [4]. Though this intervention remains safe, that does not mean acting without caution. Therefore, the performed assessment includes, among other things, the search for comorbidities and latent effects of the various treatments. The type of cancer, the often unpredictable risk of its development, and the variability of patients' reactions to the disease and therapy make it challenging to standardize rehabilitation modalities through physical activity [4]. This is why rehabilitation programs with physical activity must be individualized and constantly readjusted for some patients, following a multidisciplinary team's assessment of the patient's condition. Despite the benefits and safety of physical activity for cancer patients, several challenges remain: How do we overcome the barriers to promoting physical activity in cancer care? How do we integrate the promotion of physical activity into the cancer care system?

### Barriers to physical activity's promotion in cancer care

Healthcare professionals are in an excellent position to promote physical activity, given the trust they have from their patients and the multiple contacts they have with them during all cancer care pathways. As a result, they can participate in changing patient behaviors. Healthcare professionals' knowledge and favorable views about physical activity in cancer care positively influence their attitude towards the physical activity advice for their patients. Indeed, the high physical activity advice rate has been associated with favorable healthcare professionals' views and their sound knowledge about physical activity's benefits in cancer care [7].

The lack of knowledge about physical activity among health professionals cannot alone explain the lack of physical activity promotion. Physical activity promotion is confronted with several barriers, which can be categorized into several levels: institutional, professional, and related to the patient [8]. First, Barriers related to healthcare professionals, such as lack of information, prevent them from providing physical activity advice to patients. Secondly, Barriers related to patients, like negative perceptions and difficult access to physical activity. When we talk about the barriers related to the patient, we must not forget that this is a person who evolves in a socioeconomic and cultural environment in which family and friends play a determining role. Thirdly, institutional or structural barriers, like the lack of time and funding. For example, the absence of a rehabilitation program through physical activity in oncology hospitals could be considered a robust structural barrier. Given the barriers mentioned above, it is necessary for health system decision-makers, on the one hand, to increase training actions for health professionals in this field; and, on the other hand, to improve the cancer care system by adopting rehabilitation programs through physical exercise. The educational approach must be comprehensive, including all socioeconomic and cultural aspects of the patient, and be family-centered.

### Integration of physical activity interventions in cancer care

Physical activity remains an easily accessible intervention. Depending on the potential of the existing care system, physical activity could eventually integrate cancer care or even palliative care because it can be as much simple advice as physical exercise, which is an individualized and supervised program. Under optimal conditions, physical activity can be practiced outdoors or in a rehabilitation unit. Still, if the patient's condition requires it, it can be performed at home or even in bed.

Physical activity can take an aerobic form of exercise, such as walking. It can also be a resistance work by building muscles. Also, activities like climbing stairs or walking on a slope offer the possibility of combining the two exercise methods. In addition, balance and stretching exercises can be considered according to the patient's needs and preferences. International evidence-based guidelines have been developed for cancer patients and survivors. They have determined the modes, intensities, frequencies, and durations of physical exercise. Scientific research continues to reveal the optimal modalities of physical exercise in cancer care [5].

The physical activity advice given by health professionals should be aligned with international recommendations, such as those outlined by the Canadian Society for Exercise Physiology and the American College of Sports Medicine. Physical activity guidelines for cancer patients and survivors are now specific in duration, frequency, and intensity. Patients are asked to perform 150 minutes of moderate-intensity aerobic exercise spread over 3-5 days per week and resistance training at least two days per week. Resistance training should involve major muscles, and each session should include a warm-up and cool-down [4].

It should be noted that, in palliative care, physical activity is not conceived only on a mechanistic approach; it must be a pleasurable activity. It is about accompanying the patient in his remaining days. The patient's attention and energy must be focused on activities that are meant for him and occupations with a high capacity to reward his well-being, such as an outdoor walk to enjoy the good weather, breathing fresh air, or getting involved in community actions. The continued growth of e-health and its involvement in healthcare systems is an irreproachable asset in inactive patient behavior change. Currently, mobile applications are beginning to be used more in promoting physical activity in cancer patients, and their influence on the process of changing patient behaviors has been confirmed. A systematic review showed that mobile apps have increased daily step counts and decreased patient sedentary behavior [9]. Regarding cost, most physical activity interventions appear more cost-effective and less expensive than pharmaceutical interventions, mainly walking, exercise groups, or in-person exercise prescriptions. In contrast, the intervention of a physical exercise coach is still expensive [10]. The low cost of physical activity interventions should encourage decision-makers to facilitate their integration into the healthcare system and to make them a priority in cancer care.

**Conclusion:** physical activity is considered a supportive therapy with scientifically proven benefits and safety. Also, physical activity interventions remain less costly than pharmaceutical interventions. Healthcare systems should dismantle the abovementioned barriers to promote physical activity as a component of cancer care. Several physical activity interventions could be integrated into cancer care. Physical activity advice, therapeutic education sessions, exercise groups, and medical prescriptions are examples of less expensive and simpler forms. Using the services of a physical exercise coach still seems to be an expensive practice. The care offered must be designed with a humanist perspective and be family-centered. At first, we suggest to decision-makers to strengthen training actions in the physical activity advice to meet the needs of healthcare professionals and provide them with practical guides and educational materials for patients. After generalizing the practice of physical activity advice, it will be relevant to institute physical activity programs. The investment in rehabilitation units and multidisciplinary team training is expected to provide personalized and supervised programs. E-health is a valuable opportunity to support the process of changing patient behavior, including inactivity and a sedentary lifestyle.
